# Dexmedetomidine Attenuates Orthotopic Liver Transplantation-Induced Acute Gut Injury via *α*_2_-Adrenergic Receptor-Dependent Suppression of Oxidative Stress

**DOI:** 10.1155/2019/9426368

**Published:** 2019-11-11

**Authors:** Peibiao Lv, Tufeng Chen, Peibin Liu, Lei Zheng, Jingling Tian, Fan Tan, Jiaxin Chen, Yingqing Deng, Jun Li, Jun Cai, Xinjin Chi

**Affiliations:** ^1^Department of General Surgery, The Third Affiliated Hospital of Sun Yat-sen University, Yuedong Hospital, Meizhou, Guangdong 514700, China; ^2^Department of Gastroenterological Surgery, The Third Affiliated Hospital of Sun Yat-sen University, Guangzhou, Guangdong 510630, China; ^3^Department of Anesthesiology, The Seventh Affiliated Hospital of Sun Yat-sen University, Shenzhen, Guangdong 518017, China; ^4^Department of Anesthesiology, The Third Affiliated Hospital of Sun Yat-sen University, Guangzhou, Guangdong 510630, China

## Abstract

Patients with orthotopic liver transplantation (OLT) frequently develop acute gut injury (AGI), and dexmedetomidine (Dex) has been reported to exert a protective effect against AGI. We investigated whether Dex protects against AGI through antioxidative stress effects by the Nrf2/HO-1 antioxidative signaling pathway. Rats were randomly allocated into a sham group and six orthotopic autologous liver transplantation (OALT) groups receiving different doses of Dex together with/without *α*_2_-adrenergic receptor (AR) blockers. Intestinal tissues were collected to visualize the barrier damage and to measure the indexes of oxidative stress. For *in vitro* studies, rat intestinal recess epithelial cells (IEC-6) underwent hypoxia/reoxygenation (H/R), and the protective role of Dex was evaluated after *α*_2A_-AR siRNA silencing. OALT resulted in increased oxidative stress, significant intestinal injury, and barrier dysfunction. Dex attenuated OALT-induced oxidative stress and intestinal injury, which was abolished by the pretreatment with the nonspecific *α*_2A_-AR siRNA blocker atipamezole and the specific *α*_2A_-AR siRNA blocker BRL-44408, but not by the specific _2B/C_-AR siRNA blocker ARC239. Silencing of *α*_2A_-AR siRNA also attenuated the protective role of Dex on alleviating oxidative stress in IEC-6 cells subjected to H/R. Dex exerted its protective effects by activating Nrf2/HO-1 antioxidative signaling. Collectively, Dex attenuates OALT-induced AGI via *α*_2A_-AR-dependent suppression of oxidative stress, which might be a novel potential therapeutic target for OALT-induced AGI.

## 1. Introduction

Orthotopic liver transplantation (OLT) is considered to be the best treatment for patients with end-stage liver diseases [1]. However, inferior vena cava occlusion during OLT can induce stasis of the inferior and superior mesenteric veins, which often results in acute gut injury (AGI). Manifested by intercellular tight junction dysfunction and destruction of intestinal barriers, AGI is a life-threatening condition with a disastrous prognosis [2]. Intestinal injury contributes to a high mortality rate due to injury-associated bacterial translocation, oxidative stress, inflammatory response, and eventual multiple organ dysfunction [3, 4]. Although intestinal epithelial cell protection is considered to be crucial for patients undergoing liver transplantation, the detailed mechanisms responsible for OLT-induced AGI remain largely unknown. In addition, there is no effective treatment strategy to combat intestinal ischemia/reperfusion (IR) injury following OLT [5]. Therefore, there is an urgent need to explore the mechanisms underlying OLT-induced AGI and to develop an effective therapy for intestinal protection.

Dexmedetomidine (Dex) is an *α*_2_-adrenergic receptor (AR) agonist that is broadly used during the perioperative period to offer hemodynamic stability and an intraoperative anesthetic-sparing effect in patients [6]. Dex also has been reported to markedly reduce the inflammatory response and oxidative stress in the liver as well as other remote organs associated with hepatic IR injury [7, 8]. In addition, the protective effects of Dex have been demonstrated by the downregulation of inflammation in various types of tissue injury, such as ischemic brain injury [9], acute kidney injury [10, 11], and myocardial IR injury [12]. Of note, our previous study also has revealed that pretreatment with Dex protects rats against OLT-induced renal injury by activating *α*_2A_-AR siRNA [13, 14]. Recently, Dex administration has been reported to attenuate intestinal injury in rat models with intestinal IR [15] or cecal ligation and puncture [16]. However, whether Dex can also exert protective effects on the intestinal mucosal barrier after OLT and which subtype of *α*_2_-AR siRNA is associated with the alleviation of AGI remain unanswered. Therefore, the present study is aimed at exploring the intestinal protective effect of Dex and the role of *α*_2A_-AR siRNA in a rat model with orthotopic autologous liver transplantation (OALT) as well as the rat intestinal epithelial cell line IEC-6.

## 2. Materials and Methods

### 2.1. Animals and Treatment

Male Sprague-Dawley rats (8–10 weeks old and 220–250 g) were purchased from the Medical Experimental Animal Center of Guangzhou University of Traditional Chinese Medicine. Fifty-six rats were randomized into seven groups (*n* = 8 per group). Rats in the sham-operated group (S) did not undergo OALT. Rats in the model group (M) were intraperitoneally injected with saline 30 min before OALT. Rats in the D1 and D2 groups were intraperitoneally injected with 10 *μ*g/kg (drug/body weight) and 50 *μ*g/kg Dex (Hengrui Pharmaceutical Co., Ltd., Jiangsu, China) 30 min before OALT, respectively. Rats in the B1, B2, and B3 groups were intraperitoneally injected with 500 g/kg atipamezole (a nonspecific *α*_2A_-AR siRNA blocker, Sigma-Aldrich, USA), 50 g/kg ARC239 (a specific *α*_2B/C_-AR blocker, Santa Cruz Biotechnology, Inc., USA), and 1.5 mg/kg BRL-44408 (a specific *α*_2A_-AR siRNA blocker, Sigma-Aldrich, USA) 40 min before receiving 50 *μ*g/kg Dex prior to OALT, respectively. This study was performed with the approval of the Institutional Animal Care and Use Committee of Sun Yat-sen University in Guangzhou, China, and in accordance with the principles stated in the Guide for the Care and Use of Laboratory Animals.

### 2.2. Rat OALT Model

The OALT model was established as described previously [17]. Rats were anaesthetized, and the bile ducts, vessels, and ligaments around the liver were all dissociated. Heparin (Beijing Solarbio Science & Technology Co., Ltd., Beijing, China.) was injected via the tail vein at a dose of 200 units/kg. Four vessels, including the inferior hepatic vena cava (IHVC), superhepatic vena cava (SHVC), portal vein (PV), and hepatic artery (HA), were entirely anatomized, and a cannula was then inserted into the PV. After that, the PV and HA were clamped with atraumatic hemostatic clips, followed by occlusion of the SHVC and IHVC blood flow. The liver was then irrigated with 250 U of cold heparin at a rate of 2.0 mL/min through the PV catheter, and a 1.0 mm hole was made in the wall of the IHVC as an outflow tract. The entire time allowed for ischemia was 20 min. Finally, the openings in the PV and IHVC were repaired using 10-0 sutures; and the PV, SHVC, IHVC, and HA were unclamped. Blood samples and intestinal tissues were collected at 8 h after reperfusion.

### 2.3. Cell Culture and Treatment

The rat intestinal recess epithelial cell line IEC-6 was obtained from the American Type Culture Collection (ATCC, Manassas, USA) and was authenticated and tested as free of mycoplasma contamination by the ATCC. IEC-6 cells were cultured in Dulbecco's modified Eagle's medium (DMEM) supplemented with 10% heat-inactivated fetal bovine serum (Gibco), 100 units/mL penicillin, and 100 *μ*g/mL streptomycin (Gibco). Cells were grown at 37°C in a humidified atmosphere containing 5% CO_2_. The IEC-6 cell hypoxia/reoxygenation (H/R) model was performed to mimic intestinal IR injury during OLT in this study. The control group cells (Group A) did not undergo H/R treatment. When reaching a confluence of 70–80%, the cells in Group B (H/R) were incubated in low-glucose and serum-deprived DMEM under low-oxygen (1% O_2_) conditions for 24 h in a humidified hypoxia incubator (Thermo Fisher Scientific, USA) and then transferred back to normal oxygen conditions (95% air+5% CO_2_) for 4 h; this procedure was denoted as H24R4. The IEC-6 cells in Group C (Dex+H/R) were pretreated with 1 nM Dex for 1 h before inducing H/R injury. The cells used in Group D (siRNA), Group E (siRNA+H/R), and Group F (siRNA+Dex+H/R) were pretransfected with 50 nM siRNA against *0α*_2A_-AR siRNA. At 48 h after transfection, the cells in Group D did not receive further treatment. The cells in Groups E and Group F underwent H/R treatment, but Group F was pretreated with 1 nM Dex for 1 h.

### 2.4. Inhibition of *α*_2A_-AR Expression by siRNA Transfection

The *α*_2A_-AR siRNA duplexes (5′-CGAGCUGCAAGAUUAACGA-3′) targeting the rat *α*_2A_-AR siRNA gene (gene ID: 25083; nucleotide accession number: NM_012739.3) were commercially obtained from Biomics (Jiangsu, China). A nonspecific siRNA was used as the control siRNA (NC group). The efficiency of siRNA transfection was tested using a fluorescent-labeled siRNA. IEC-6 cells at 50% confluence were incubated with *α*_2A_-AR siRNA (50 nM) for 48 h before they were subjected to H24R4 treatment. The transfection of IEC-6 cells was carried out using the transfection reagent Lipofectamine® 3000 (Life Technologies) according to the manufacturer's instructions.

### 2.5. Histopathological Examination

Intestinal tissues were obtained from the site at 5 cm above the terminal ileum. After being embedded in paraffin, these tissues were cut into 5 *μ*m thick samples for hematoxylin and eosin staining. The intestinal mucosal damage was graded according to Chiu's criteria [18]: Grade 0—normal mucosa villi; Grade 1—development of the subepithelial Gruenhagen's space at the tip of the villi; Grade 2—extension of the subepithelial space with moderate epithelial lifting; Grade 3—extensive epithelial lifting, possibly with a few denuded villi; Grade 4—denuded villi with dilated capillaries as well as increased cellularity of the lamina propria and exposed capillaries; and Grade 5—disintegration of the lamina propria, ulceration, and hemorrhage.

### 2.6. Enzyme-Linked Immunosorbent Assay (ELISA)

Blood samples were drawn from the inferior vena cava at 8 h after OLT. After clotting at room temperature for 30 min, the blood was centrifuged (2500 rpm at 4°C for 20 min) to isolate the serum. Intestinal tissue was transformed into 10% homogenates with frozen normal saline and spun at 3000 rpm for 10 min. The pulmonary protein content was measured using a Bicinchoninic Acid Protein Assay Kit (Nanjing KeyGen Biotech Co., Ltd., Nanjing, China). The concentrations of serum biomarkers of intestinal mucosal barrier function, including diamine oxidase (DAO), lipopolysaccharide (LPS), intestinal fatty acid-binding protein 2 (I-FABP2), and D-lactate (D-LA); intestinal antioxidants, including superoxide dismutase (SOD), glutathione S-transferase *Α*1 (GST*α*1), and glutathione (GSH); and inflammatory factors, including interleukin- (IL-) 1*β* and tumor necrosis factor-*α* (TNF-*α*) in intestinal tissue, were measured using commercially available ELISA kits (Wuhan USCN Business Co., Ltd., Hubei, China) according to the protocols provided by the manufacturer. The absorbance at 450 nm (OD 450) was determined using a microplate reader (BioTek, MQX200).

### 2.7. Detection of Reactive Oxygen Species (ROS)

As reported previously [19], the accumulation of ROS in the intestinal tissue was estimated by using the *in vitro* reactive nitrogen species assay kit OxiSelect (Cell Biolabs Inc., San Diego, CA, USA). Briefly, equal protein amounts from isolated rat intestinal homogenates were resuspended and subsequently added to wells of a 96-well plate suitable for fluorescence measurement. Catalyst and DCFH solution were added according to the manufacturer's protocol. The relative fluorescence was read using a fluorescence plate reader at 480 nm excitation/530 nm emission.

### 2.8. Cell Viability Assay

IEC-6 cells were plated in 96-well plates at a density of 5000 cells/well. After the specified stimulations, the *in vitro* cell viability was tested using a Cell Counting Kit-8 assay (Nanjing KeyGen Biotech Co., Ltd.) according to the manufacturer's instructions. Briefly, the IEC-6 cells plated in 96-well plates were cocultured with the CCK-8 reagents at 37°C for 30 minutes, and the relative fluorescence was measured at 450 nm excitation using a microplate reader (BioTek, MQX200).

### 2.9. Flow Cytometry

Cells were collected at 4 h after reoxygenation. For analysis of apoptosis, cells were stained by annexin V-FITC and counterstained with propidium iodide, and then analyzed by flow cytometry according to the manufacturer's instructions (Nanjing KeyGen Biotech Co., Ltd.). Data analysis was conducted by FlowJo 7.6 software (FlowJo, LLC).

### 2.10. Quantitative Real-Time Polymerase Chain Reaction (qRT-PCR)

Total RNA was extracted from IEC-6 cells using the TRIzol Reagent (Invitrogen). Reverse transcription was performed using ReverTra Ace qPCR RT Master Mix (TOYOBO, Japan). Quantitative analysis of target genes, including IL-1*β*, TNF-*α*, heme oxygenase-1 (HO-1, known as HMOX1 for gene name), SOD, and catalase (CAT), was conducted using SYBR® Green Realtime PCR Master Mix (TOYOBO, Japan) with Roche LightCycler 1.1. *β*-Actin was used as the housekeeping gene. The primer sequences are listed in Table 1. All samples were tested in quadruplicate, and the differences of threshold cycles (CT) between target genes and *β*-actin were normalized to that of the sham group according to the ΔΔCT method [20].

### 2.11. Immunofluorescence

Cells were plated onto sterile cover slips and then subjected to the H/R protocol. Samples were stained with antibodies against nuclear factor- (NF-) *κ*B, toll-like receptor- (TLR-) 4, and nuclear factor erythroid 2-related factor 2 (Nrf2) (Santa Cruz Biotechnology, Inc.) at 4°C overnight, followed by staining with anti-rabbit or anti-mouse IgG-FITC (1 : 1000, Millipore) antibody according to the manufacturer's instructions. Nuclei were stained with 4′,6-diamidino-2-phenylindole (1 *μ*g/mL; Nanjing KeyGen Biotech Co., Ltd.). All images were measured using an EVOS FL fluorescence microscope (EVOS FL, Life Technologies).

### 2.12. Western Blot Analysis

Western blotting was performed in accordance with standard procedures, as described previously [2]. Anti-occludin, anti-zonula occludens-1 (ZO-1), anti-Nrf2, anti-NQO1, and anti-Keap1 (Santa Cruz Biotechnology, Inc.) antibodies and secondary antibody (1 : 2000; Millipore) were used to detect targeted protein expression. Anti-*β*-actin antibody (Proteintech) was used at 1 : 5000. Images were acquired by a Tanon 5500 imaging system (Tanon, Shanghai). ImageJ software was used to quantitate the staining intensity in the images, and the data were normalized to the sham or control values.

### 2.13. Statistical Analysis

SPSS 13.0 (SPSS Inc., Chicago, IL, USA) and SigmaPlot 10.0 (Systat Software, Inc., Chicago, IL, USA) were used to perform statistical analysis. Normality of the data was tested using the Kolmogorov-Smirnov test. Multiple comparisons among different groups were analyzed using one-way analysis of variance, followed by Tukey's post hoc test. Quantitative data are presented as means ± standard deviation. *P* values less than 0.05 were considered statistically significant.

## 3. Results

### 3.1. Blocking *α*_2A_-AR siRNA Reversed the Protective Role of Dex on OALT-Induced Intestinal Injury in Rats

**Figure 1:**
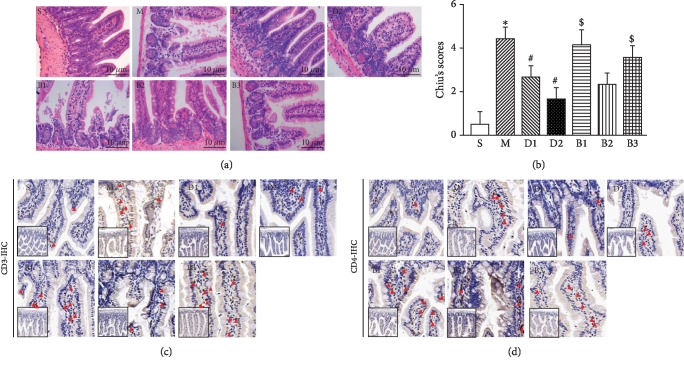
Blocking *α*_2A_-AR siRNA reversed the protective role of Dex on OLT-induced intestinal injury in rats. (a) Representative images showing the microscopic findings of intestines stained with hematoxylin and eosin in seven groups. Bar scale, 10 *μ*m. (b) Chiu's scoring quantitating the intestinal mucosal damage. (c–d) Immunohistochemistry staining of CD3 (c) and CD4 (d) in intestinal issues. Red arrow: positive cells. Data are represented as the mean ± standard deviation, *n* = 8 for each group. S: sham-operated group; M: the model group with OLT; D1: rats that were pretreated with 10 *μ*g/kg before OLT; D2: rats that were pretreated with 50 *μ*g/kg before OLT; B1: rats that received 500 g/kg atipamezole at 40 min before receiving 50 *μ*g/kg Dex prior to OLT; B2: rats that received 50 g/kg ARC239 at 40 min before receiving 50 *μ*g/kg Dex prior to OLT; B3: rats that received 1.5 mg/kg BRL-44408 at 40 min before receiving 50 *μ*g/kg Dex prior to OLT. ^∗^*P* < 0.05, compared to Group S; ^#^*P* < 0.05, compared to Group M; ^$^*P* < 0.05, compared to Group D2.

The rat OALT model was first established to test the protective role of Dex on AGI. As shown in Table 2, the rat body weights and the times of the warm ischemia phase among the seven groups were comparable, thus excluding the effect of internal variation on intestinal pathological analysis. While the intestinal mucosa was integrated and the epithelium was compactly arrayed in the sham-operated rats (Group S), the OALT model rats (Group M) had significantly damaged intestinal glands, mucosal villi edema (Figure 1(a)), and neutrophil infiltration, as marked by CD3 and CD4 (Figures 1(c) and 1(d)). Compared with Group M, the pathological damage of the ileum was dose-dependently ameliorated in rats pretreated with Dex (Group D1 and Group D2), as evidenced by an increased villi height and reduced neutrophil infiltration in Group D2 (50 *μ*g/kg) compared to Group D1 (10 *μ*g/kg). Compared to Group D2, pretreatment with atipamezole (a nonspecific *α*_2A_-AR siRNA blocker, Group B1) and BRL-44408 (a specific *α*_2A_-AR siRNA blocker, Group B3) in rats significantly reversed the protective effect of Dex, whereas there was no evident effect in rats treated with ARC329 (a specific *α*_2B/C_-AR blocker, Group B2) (Figure 1(a)). As shown in Figure 1(b), Chiu's scoring, which quantitated intestinal mucosal damage, indicated that either the nonspecific or specific *α*_2A_-AR siRNA blocker, but not the *α*_2B/C_-AR siRNA blocker, reversed the protective role of Dex on OALT-induced intestinal injury in rats.

### 3.2. Blocking *α*_2A_-AR siRNA Reversed the Protective Role of Dex on OALT-Induced Intestinal Mucosal Barrier Injury in Rats

**Figure 2:**
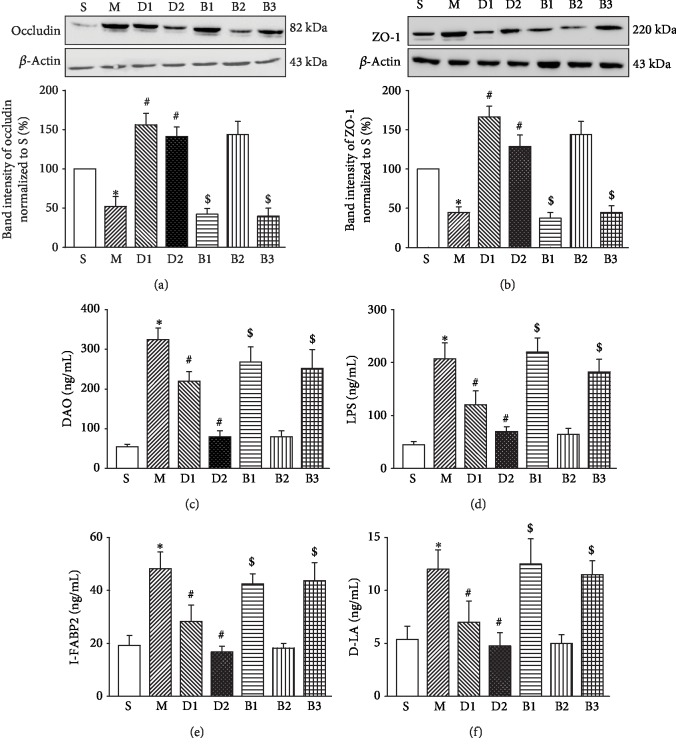
Blocking *α*_2A_-AR siRNA reversed the protective role of Dex on OLT-induced intestinal mucosal barrier injury in rats. (a–b) Western blotting results showing the levels of occludin (a) and ZO-1 (b) in rat intestines. (c–f) ELISA results showing the levels of serum biomarkers of intestinal mucosal barrier function, including DAO (c), LPS (d), I-FABP2 (e), and D-LA (f). Data are represented as the mean ± standard deviation, *n* = 8 for each group. S: sham-operated group; M: the model group with OLT; D1: rats that were pretreated with 10 *μ*g/kg before OLT; D2: rats that were pretreated with 50 *μ*g/kg before OLT; B1: rats that received 500 g/kg atipamezole at 40 min before receiving 50 *μ*g/kg Dex prior to OLT; B2: rats that received 50 g/kg ARC239 at 40 min before receiving 50 *μ*g/kg Dex prior to OLT; B3: rats that received 1.5 mg/kg BRL-44408 at 40 min before receiving 50 *μ*g/kg Dex prior to OLT. ^∗^*P* < 0.05, compared to Group S; ^#^*P* < 0.05, compared to Group M; ^$^*P* < 0.05, compared to Group D2.

Next, we investigated the alterations of tight junction molecules in the rat intestinal mucosa, which can be used to indicate dysfunction of the intestinal mucosal barrier. The Western blotting results showed a significant reduction of occludin (Figure 2(a), original images in [Supplementary-material supplementary-material-1]) and ZO-1 (Figure 2(b), original images in [Supplementary-material supplementary-material-1]) protein expression in Group M, compared to Group S. Additionally, serum biomarkers of intestinal mucosal barrier function, including DAO (Figure 2(c)), LPS (Figure 2(d)), I-FABP2 (Figure 2(e)), and D-LA (Figure 2(f)), were all increased in Group M compared to Group S, suggesting significant damage of the rat intestinal mucosal barrier. Compared to Group M, Group D1 and Group D2 (pretreated with Dex) had significantly increased expression of occludin and ZO-1 as well as remarkably reduced serum DAO, LPS, I-FABP2, and D-LA levels (*P* < 0.05). Compared with Group D2, the expression levels of occludin and ZO-1 were significantly reduced in Group B1 and Group B3, and the concentrations of serum DAO, LPS, I-FABP2, and D-LA were all significantly increased in Group B1 and Group B3 (*P* < 0.05). However, Group B2 had comparable levels of all the parameters tested to Group D2 (all *P* > 0.05).

### 3.3. Blocking *α*_2A_-AR siRNA Counteracted the Alleviation of Oxidative Stress by Dex in Intestines of Rats with OALT

**Figure 3:**
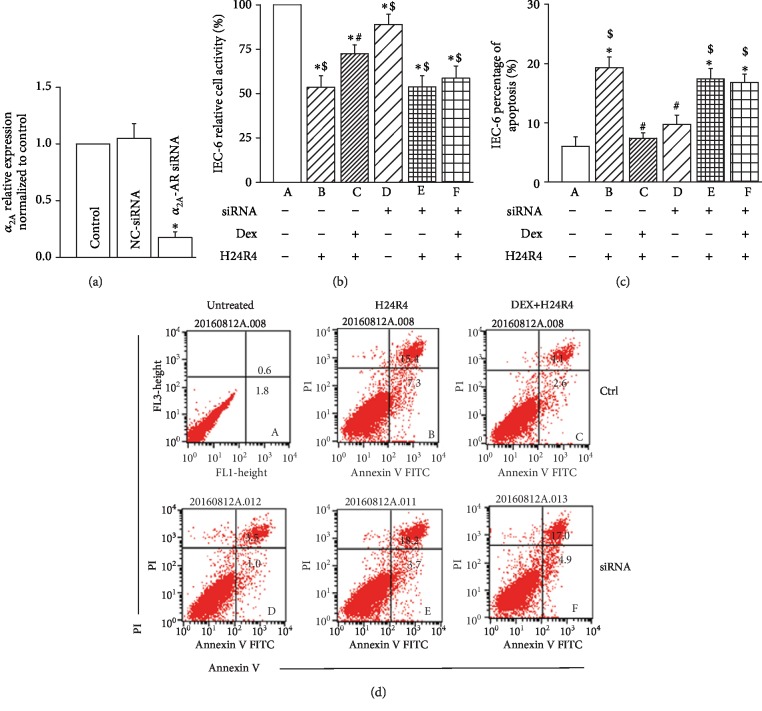
Blocking *α*_2A_-AR siRNA counteracted the alleviation of oxidative stress by Dex in intestines of rats with OLT. The levels of oxidant ROS (a) and antioxidants including GST*α*1 (b), SOD (c), and GSH (d) were measured in rat intestines. Data are represented as the mean ± standard deviation, *n* = 8 for each group. S: sham-operated group; M: the model group with OLT; D1: rats that were pretreated with 10 *μ*g/kg before OLT; D2: rats that were pretreated with 50 *μ*g/kg before OLT; B1: rats that received 500 g/kg atipamezole at 40 min before receiving 50 *μ*g/kg Dex prior to OLT; B2: rats that received 50 g/kg ARC239 at 40 min before receiving 50 *μ*g/kg Dex prior to OLT; B3: rats that received 1.5 mg/kg BRL-44408 at 40 min before receiving 50 *μ*g/kg Dex prior to OLT. ^∗^*P* < 0.05, compared to Group S; ^#^*P* < 0.05, compared to Group M; ^$^*P* < 0.05, compared to Group D2.

To further explore the mechanism underlying the protective effect of Dex on OALT-induced AGI, we also measured the oxidants and antioxidants in rat intestines. As shown in Figure 3, the levels of ROS were significantly increased in Group M compared to Group S (*P* < 0.05). Meanwhile, the activity of antioxidants, including GST*α*1, GSH, and SOD, were all suppressed in Group M compared with Group S (*P* < 0.05). In Group D1 and Group D2 (pretreated with Dex) as well as Group B2 (pretreated with Dex and ARC239), the levels of ROS were decreased and the activities of the enzymes, including GST*α*1, GSH, and SOD, were significantly increased, compared to those of Group M (*P* < 0.05). The protective effects of Dex were reversed by treatment with either atipamezole (Group B1) or BRL-44408 (Group B3) (*P* < 0.05), but not ARC239 (Group B2).

### 3.4. Silencing of *α*_2A_-AR siRNA Reversed the Protective Role of Dex on Cell Apoptosis in IEC-6 Cells with Simulated H/R Stimulation

**Figure 4:**
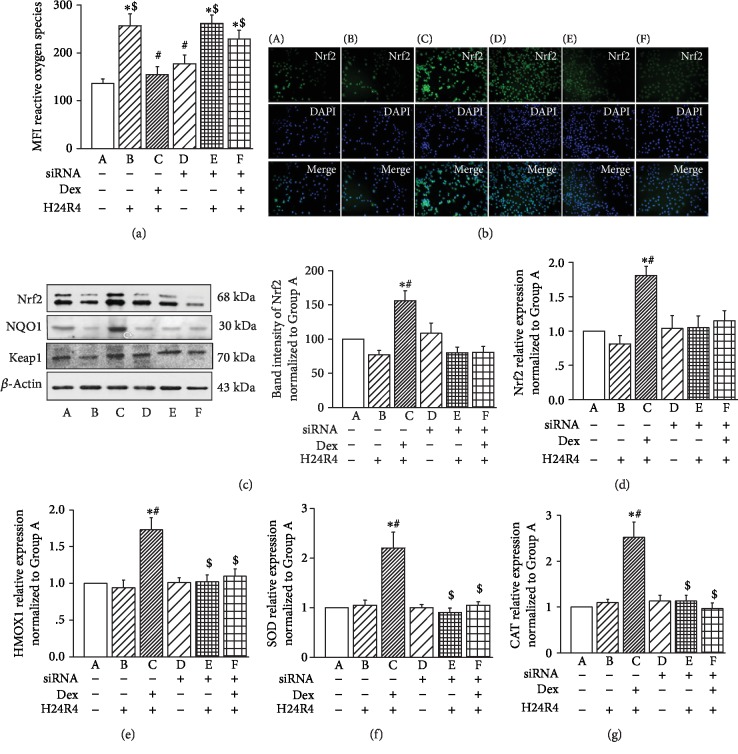
Silencing of *α*_2A_-AR siRNA reversed the protective role of Dex on cell apoptosis in IEC-6 cells with simulated H/R stimulation. (a) qRT-PCR data showing effective knockdown of *α*_2A_-AR by siRNA transfection in IEC-6 cells. Control: untreated IEC-6 cells; NC-siRNA: IEC-6 cells transfected with nonspecific control siRNA; *α*_2A_-AR siRNA: IEC-6 cells transfected with *α*_2A_-AR-specific siRNA. (b) Results of the cell proliferation assay by Cell Counting Kit-8 in IEC-6 cells with different stimulations. (c) Summary of cell apoptosis data of IEC-6 cells with different stimulations. (d) Representative flow cytometry profiles showing the cell apoptosis assay by annexin V and propidium iodide staining, *n* = 6 for each group. A: control IEC-6 cells; B: IEC-6 cells with H/R treatment; C: IEC-6 cells that were pretreated with 1 nM Dex for 1 h before inducing H/R injury; D: IEC-6 cells with silencing of *α*_2A_-AR siRNA; E: IEC-6 cells with silencing of *α*_2A_-AR siRNA and H/R treatment; F: IEC-6 cells with silencing of *α*_2A_-AR siRNA that were pretreated with Dex prior to H/R injury. ^∗^*P* < 0.05, compared to Group A; ^#^*P* < 0.05, compared to Group B; ^$^*P* < 0.05, compared to Group C.

To confirm that Dex attenuated OALT-induced rat intestinal injury through suppressing oxidative stress, we established an H24R4 model with the intestinal recess epithelial cell line IEC-6. In this model, IEC-6 cells were stressed under hypoxic conditions for 24 h and returned to normoxic conditions for another 4 h, mimicking the H/R stimulation in rats with OALT. We also knocked down *α*_2A_-AR by siRNA transfection in IEC-6 cells (Figure 4(a); the transfection efficiency is shown in [Supplementary-material supplementary-material-1]) to test the role of *α*_2A_-AR siRNA on the protective effects of Dex. Compared with the control IEC-6 cells (Group A), siRNA knockdown of *α*_2A_-AR siRNA alone (Group D) did not affect cell proliferation (Figure 4(b)) or cell apoptosis (Figure 4(c)). In addition, H24R4-induced cell damage (Group B) was markedly attenuated (*P* < 0.05) when IEC-6 cells were pretreated with Dex in Group C. However, as also shown in the representative flow cytometry profile (Figure 4(d)), the effect of Dex on attenuating cell apoptosis was erased when *α*_2A_-AR siRNA was silenced, as no significant changes could be observed between Group E (without Dex pretreatment) and Group F (with Dex pretreatment). Moreover, the effect of Dex was suppressed when *α*_2A_-AR siRNA was functionally inhibited through *α*_2A_-AR-siRNA-mediated silencing (*P* < 0.05, Group F vs. Group C).

### 3.5. Silencing of *α*_2A_-AR siRNA Erased the Protective Role of Dex on Antioxidation in IEC-6 Cells with Simulated H/R Stimulation

**Figure 5:**
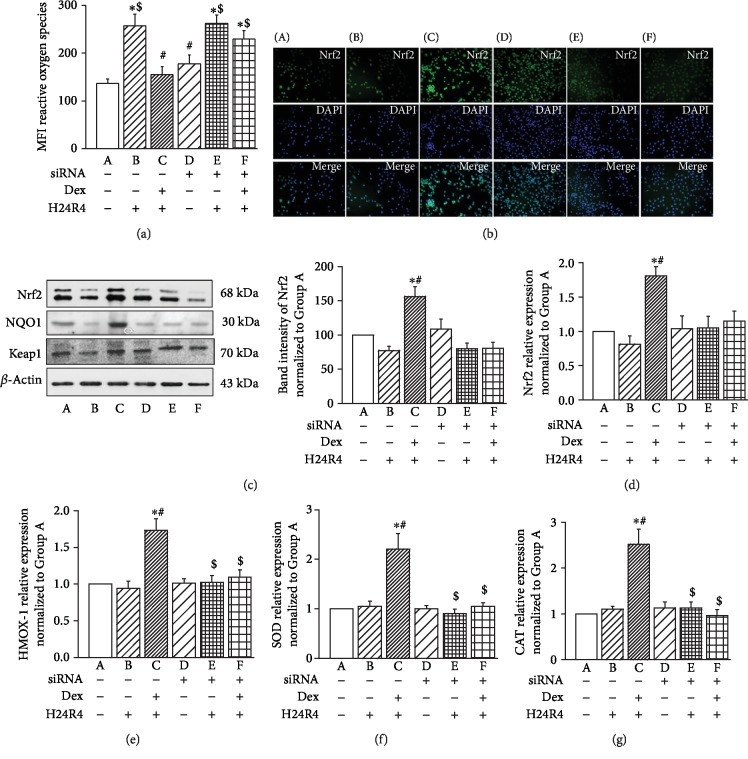
Silencing of *α*_2A_-AR siRNA erased the protective role of Dex on antioxidation in IEC-6 cells with simulated H/R stimulation. (a) Summary of ROS levels in IEC-6 cells with different treatments. (b) Representative fluorescence immunostaining images of Nrf2 in IEC-6 cells with different treatments. Scale bar, 100 *μ*m. (c–d) Expression of Nrf2, NQO1, and Keap1 proteins and mRNA levels in intestinal tissues. (e–g) Summary of the relative expression levels of the transcripts for the genes HMOX1 (e), SOD1 (f), and CAT (g) in IEC-6 cells with different treatments, *n* = 6 for each group. A: control IEC-6 cells; B: IEC-6 cells with H/R treatment; C: IEC-6 cells that were pretreated with 1 nM Dex for 1 h before inducing H/R injury; D: IEC-6 cells with silencing of *α*_2A_-AR siRNA; E: IEC-6 cells with silencing of *α*_2A_-AR siRNA and H/R treatment; F: IEC-6 cells with silencing of *α*_2A_-AR siRNA that were pretreated with Dex prior to H/R injury. ^∗^*P* < 0.05, compared to Group A; ^#^*P* < 0.05, compared to Group B; ^$^*P* < 0.05, compared to Group C.

The ROS levels in the IEC-6 cells of Group B were significantly increased after H24R4 stimulation compared to that of Group A (*P* < 0.05, Figure 5(a)). While the ROS level was inhibited by Dex treatment in Group C, knockdown of *α*_2A_-AR by siRNA transfection substantially abolished the effect of Dex, as demonstrated by largely comparable ROS levels between Group E and Group F (Figure 5(a)). Since Nrf2 is a transcriptional factor that exhibits an antioxidative effect by increasing the levels of antioxidants, such as SOD and CAT, we investigated whether Dex exerts its effects through the Nrf2/HO-1 signaling pathway. Immunofluorescence staining, qPCR, and Western blot all revealed that the Nrf2 protein was at basic expression levels in Group A and Group D (Figure 5(b)). H24R4 treatment significantly inhibited the expression and nuclear translocation of Nrf2 in IEC-6 cells in Group B and Group E. The additional pretreatment with Dex upregulated the expression of Nrf2 in Group C, but not in Group F (Figures 5(b)–5(d)), suggesting that the effect of Dex is dependent on *α*_2A_-AR siRNA. Accordingly, the expression patterns of the transcripts for the genes *HO-1* (Figure 5(c)), *SOD* (Figure 5(d)), and *CAT* (Figure 5(e)) were all consistent with that of *Nrf2*.

## 4. Discussion

Oxidative stress plays an important role in the progression of AGI [21]. Therefore, the inhibition of oxidants effectively promotes intestinal barrier function [22]. Dex contributes to a variety of health-promoting activities, including antiapoptotic and antioxidant activities [13, 14]. In the current study, we demonstrated that pretreatment of rats with Dex dose-dependently reduced injury of the intestinal mucosal barrier during OALT-induced AGI. We further demonstrated that these beneficial effects of Dex were abolished by the nonspecific *α*_2A_-AR siRNA blocker atipamezole and the *α*_2A_-AR-specific blocker BRL-44408, but not the *α*_2B/C_-AR siRNA-specific blocker ARC239. Furthermore, we showed that blocking *α*_2A_-AR siRNA counteracted the alleviation of oxidative stress by Dex in the intestines of rats with OALT. Moreover, Dex attenuated H/R-induced suppression of the Nrf2 pathway in IEC-6 cells, while these effects of Dex were reduced or abolished by *α*_2A_-AR siRNA silencing. These findings together suggest that Dex could effectively attenuate OALT-induced AGI through inhibiting oxidative stress to alleviate intestinal mucosal barrier injury, at least in part, via targeting *α*_2A_-AR siRNA.

Inferior vena cava occlusion during OLT is associated with intestinal IR injury, which may result in gut barrier failure and ultimately a systemic inflammatory state. It also has been demonstrated that oxidative damage to the intestinal mucosal layer is a critical component of gut barrier failure [23]. In addition, Dex has been reported to protect against intestinal epithelial barrier disruption in endotoxemic rats and patients who underwent laparoscopic resection of colorectal cancer [24, 25]. Moreover, Xia et al. [26] have reported that Dex protects against heart stroke-induced multiorgan dysfunction via maintaining the intestinal integrity. Consistent with these reports, we found that OALT led to significant gut barrier failure, as reflected by intestinal epithelial cell injury and weakened tight junctions (demonstrated by the protein expression of occludin and ZO-1), as well as the upregulation of multiple specific biomarkers related to gut barrier dysfunction, including DAO, LPS, I-FABP2, and D-LA. We also found that pretreatment with Dex significantly protected the gut barrier function against OALT, which could be reversed by a nonspecific *α*_2A_-AR siRNA blocker and a specific *α*_2A_-AR siRNA blocker.

During the process of OALT, intestinal congestion and IR induce injury to the mucosal barrier, which increases the mucosal permeability and subsequently promotes the overgrowth and translocation of local bacteria. This results in oxidative stress and leads to AGI. In previous studies, Dex administration has been reported to protect against oxidative stress in other organs that had undergone OALT [13, 17, 18]. Consistent with these results, in the present study, we found that pretreatment with Dex attenuated AGI by reducing intestinal oxidative stress both *in vivo* and *in vitro* in a rat model and in IEC-6 cells, respectively.

Importantly, we found that pretreatment with Dex significantly inhibited ROS accumulation by activating the Nrf2/HO-1 signaling pathway. It is well known that excessive ROS production is one of the main mechanisms involved in intestinal IR injury, whereas antioxidant enzymatic activity alleviates AGI induced by IR [27]. Acting like a transcriptional factor and cell defense response regulator, Nrf2 translocates into the nucleus following exposure to oxidative stress and activates the gene expression of many antioxidases and cytoprotective proteins, including GCL, HO-1, GPX, and NQO1 [28]. Recently, several studies have documented that the upregulation of Nrf2/HO-1 plays a critically protective role in AGI induced by traumatic brain injury or intestinal IR [29, 30]. However, no studies have yet reported that Dex protects against intestinal injury induced by OALT by regulating the Nrf2/HO-1 pathway and its downstream antioxidant enzymes. Similar to previous reports, we demonstrated that OALT induced significant oxidative stress by producing large amounts of ROS in the intestines. However, Nrf2 expression did not exhibit a significant increase until the ROS levels and intestinal pathological damage reached a peak at 8 h after OALT (data not shown). Interestingly, pretreatment with Dex significantly activated the Nrf2 pathway, promoted the expression of antioxidant enzymes, and protected the intestine from oxidative injury in a timely manner. Considering the concurrent occurrence of both attenuated oxidative stress and the induction of Nrf2 after Dex pretreatment, we assumed that Dex exerts its intestinal protective effect at least partly via the upregulation of the Nrf2/HO-1 signaling pathway after OALT.

There are three *α*_2_-AR subtypes. Previous studies [31, 32] have shown that *α*_2_ agonist-induced sedation, analgesia, hypotension, and hypothermia are mediated by the *α*_2A_-subtype; *α*_2_ agonist-induced startle reflex, stress response, and locomotion are mediated by the *α*_2C_-subtype; and the *α*_2_ agonist-induced hypertensive and peripheral hyperalgesic effect of norepinephrine is mediated by the *α*_2B_-subtype. Dexmedetomidine is a highly specific *α*_2_-adrenoceptor agonist with high affinity to each of the *α*2-adrenoceptor subtypes. In our present study, we focused on the classical antioxidant pathway and we did find that Dex affected this pathway in OALT-induced AGI, which has been confirmed by using the IEC-6 cell line to show that dexmedetomidine directly upregulates the Nrf2/HO-1 signaling pathway mostly via the *α*_2A_-adrenoceptor subtype.

However, there are some limitations in this current study. A further study incorporating the use of gene knockout mice for Nrf2 or HO-1 should be performed in order to confirm the role of Nrf2/HO-1 pathway upregulation in dexmedetomidine-mediated attenuation of OALT-induced AGI. The effect of posttreatment with dexmedetomidine was not determined. In addition, single cell images at higher magnification to differentiate between a nuclear and cytoplasmic staining of Nrf2 were not provided, and some new indexes of gut barrier functions, such as glucagon-like peptide-2, a calcitonin precursor, were not detected. Further research is needed to explore these areas.

In conclusion, the current study demonstrated that Dex exerts protective effects against AGI following OLT in an *α*_2A_-AR-dependent way. These protective effects are achieved through activating Nrf2/HO-1 signaling to reduce oxidative stress. Our investigation not only suggests that pretreatment with Dex represents a useful method for reducing the intestinal damage caused by liver transplantation, but it also implies the critical roles of controlling oxidative stress in the treatment of AGI.

## Figures and Tables

**Table 1:** List of the primer sequences used in this study.

Gene	Primer sequence
HMOX-1-F	5′-CAGAGTTTCTTCGCCAGAGG-3′
HMOX-1-R	5′-TGAGTGTGAGGACCCATCG-3′
SOD1-F	5′-GCAGAAGGCAAGCGGTGAAC-3′
SOD1-R	5′-TAGCAGGACAGCAGATGAGT-3′
CAT-F	5′-TATTGCCGTCCGATTCTC-3′
CAT-R	5′-ATGCCCTGGTCAGTCTTG-3′
*β*-Actin-F	5′-AGAGGGAAATCGTGCGTGAC-3′
*β*-Actin-R	5′-CCATACCCAGGAAGGAAGGCT-3′

**Table 2:** Rat body weights and times of the warm ischemia phase.

Group	Weight (g)	Time of warm ischemia (min)
S	230.9 ± 15.1	—
M	236.3 ± 19.2	19.5 ± 1.7
D1	228.8 ± 13.2	20.1 ± 1.2
D2	235.0 ± 18.2	21.0 ± 1.5
B1	233.4 ± 20.0	20.5 ± 1.1
B2	229.2 ± 15.3	20.4 ± 1.4
B3	235.1 ± 14.6	20.2 ± 1.5

Data are represented as the mean ± standard deviation, *n* = 8 for each group. S: sham-operated group; M: the model group with OLT; D1: rats that were pretreated with 10 *μ*g/kg before OLT; D2: rats that were pretreated with 50 *μ*g/kg before OLT; B1: rats that received 500 g/kg atipamezole at 40 min before receiving 50 *μ*g/kg Dex prior to OLT; B2: rats that received 50 g/kg ARC239 at 40 min before receiving 50 *μ*g/kg Dex prior to OLT; B3: rats that received 1.5 mg/kg BRL-44408 at 40 min before receiving 50 *μ*g/kg Dex prior to OLT.

## Data Availability

Readers can ask for the data in our study by sending email to chixinjin@yeah.net. We will share the data underlying the findings to the researchers who are interested in our study.

## Supplementary Materials

Supplementary Materials Figure S1: entire blot lanes including molecular weight markers for all cropped Western blot bands shown in the main body of the manuscript. The lanes in the rectangle are the exact ones in the main figure. Figure S2: efficiency of siRNA transfection in IEC-6 cells. Six hours after transfection of fluorescent-labeled NC-siRNA-FAM, the cells were observed under a fluorescence microscope. The IEC-6 cells which were successfully transfected would release green fluorescence and those unsuccessfully transfected would release no green fluorescence. Red arrow: successfully transfected cells. White arrow: unsuccessfully transfected cells.
