# Supplementary material for: Dexmedetomidine Attenuates Orthotopic Liver Transplantation-Induced Acute Gut Injury via α2-Adrenergic Receptor-Dependent Suppression of Oxidative Stress
Source: Oxid Med Cell Longev. 2019 Nov 11;2019:9426368. doi: 10.1155/2019/9426368 (PMC6885230; doi:10.1155/2019/9426368)
Supplement: Supplementary Materials — Figure S1: entire blot lanes including molecular weight markers for all cropped Western blot bands shown in the main body of the manuscript. The lanes in the rectangle are the exact ones in the main figure. Figure S2: efficiency of siRNA transfection in IEC-6 cells. Six hours after transfection of fluorescent-labeled NC-siRNA-FAM, the cells were observed under a fluorescence microscope. The IEC-6 cells which were successfully transfected would release green fluorescence and those unsuccessfully transfected would release no green fluorescence. Red arrow: successfully transfected cells. White arrow: unsuccessfully transfected cells. [file 9426368.f1.docx]

**Dexmedetomidine Attenuates Orthotopic Liver Transplantation-Induced Acute** **Gut Injury via α_2_-Adrenergic Receptor-dependent Suppression of Oxidative Stress**

Peibiao Lv^1^*; Tufeng Chen^2^*; Peibin Liu^3^*; Lei Zheng^3^; Jingling Tian^3^; Fan Tan^3^; Jiaxin Chen^3^; Yingqing Deng^3^; Jun Li^1+^; Jun Cai^4+^; Xinjin Chi^3,4+^;

^1^Department of General Surgery, the Third Affiliated Hospital of Sun Yat-Sen University, Yuedong Hospital. Meizhou, Guangdong, 514700, P.R. China.

^2^Department of Gastroenterological Surgery, The Third Affiliated Hospital of Sun Yat‑sen University, Guangzhou, Guangdong 510630, P.R. China

^3^Department of Anesthesiology, The Seventh Affiliated Hospital of Sun Yat‑sen University, Shenzhen, Guangdong 518017, P.R. China

^4^Department of Anesthesiology, The Third Affiliated Hospital of Sun Yat‑sen University, Guangzhou, Guangdong 510630, P.R. China


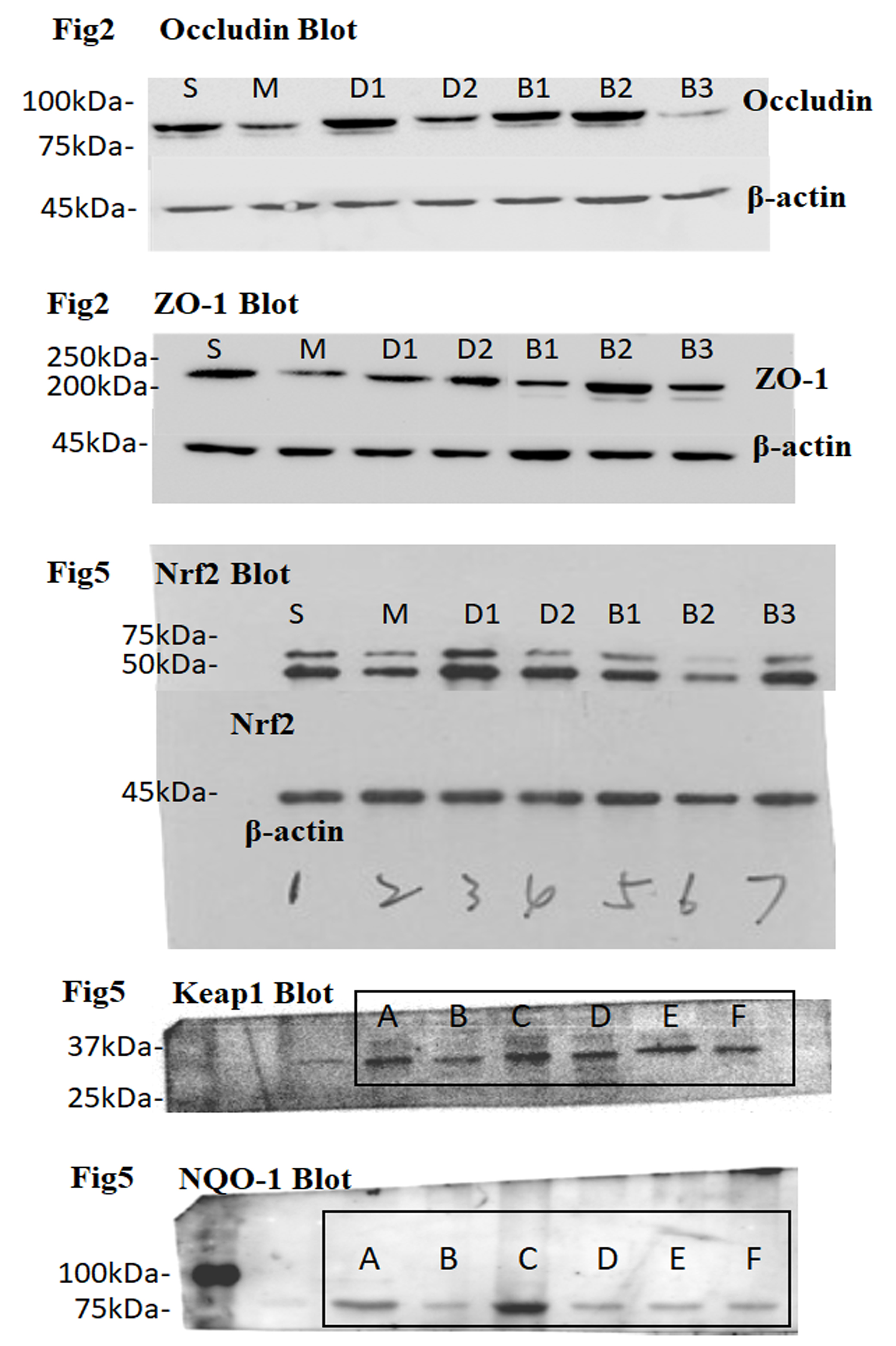


**Figure S1** **Entire blot lanes including molecular weight markers for all cropped Western blot bands shown in the main body of the manuscript.** The lanes in the rectangle are the exact ones in the main figure.


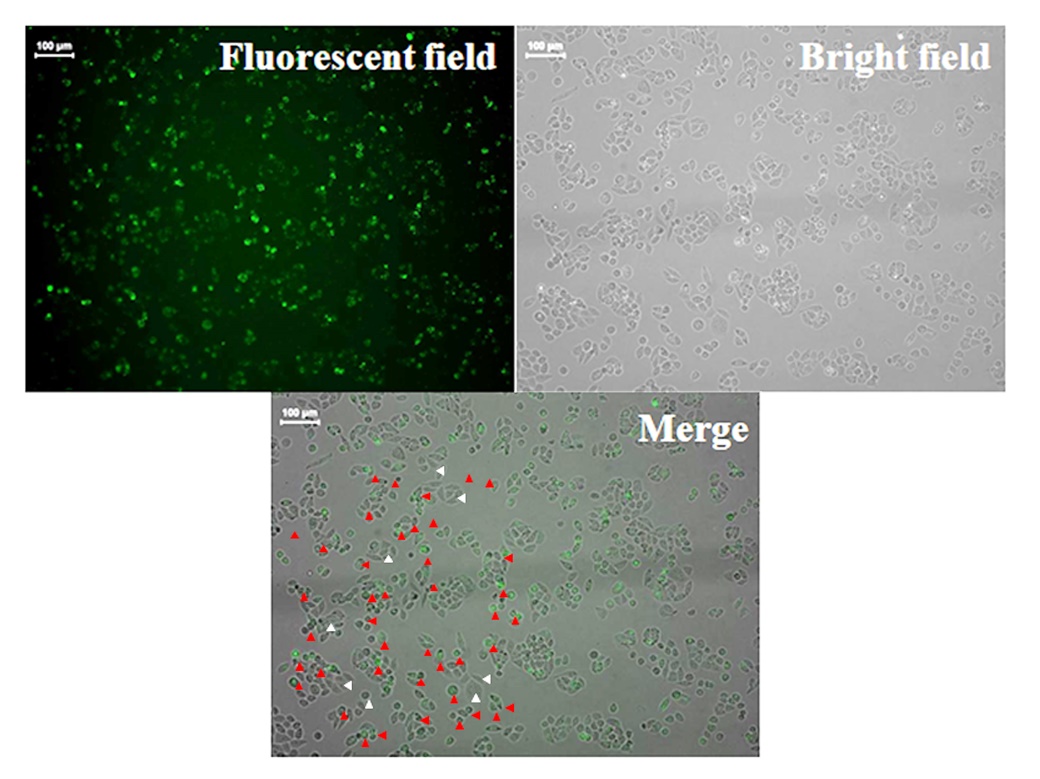


**Figure S2 Efficiency of siRNA transfection in IEC-6 cells.** Six hours after transfection of fluorescent labeled NC siRNA-FAM, the cells were observed under fluorescence microscope. The IEC-6 cells which successfully transfected would release green fluorescence and those unsuccessfully transfected release no green fluorescence. Red arrow: successfully transfected cells; White arrow: unsuccessfully transfected cells.
